# The influence of visual attention on letter recognition and reading acquisition in Arabic

**DOI:** 10.3389/fpsyg.2025.1628051

**Published:** 2025-10-17

**Authors:** Alaa Ghandour, Emmanuel Trouche, Dominique Guillo, Sylviane Valdois

**Affiliations:** ^1^Univ. Grenoble Alpes, Univ. Savoie Mont Blanc, CNRS, LPNC, Grenoble, France; ^2^Africa Institute for Research in Economics and Social Sciences, Mohammed VI Polytechnic University, Rabat, Morocco; ^3^Groupe d’Etudes des Méthodes de l’Analyse Sociologique de la Sorbonne, UMR 8598, CNRS, Sorbonne Université, Paris, France

**Keywords:** reading acquisition, Arabic language, visual attention span, letter knowledge, graphic complexity, phonological awareness, beginning readers

## Abstract

**Introduction:**

The involvement of phonological awareness (PA), rapid automatized naming (RAN) and letter knowledge (LK) in Arabic reading achievement is well established, but evidence for a unique contribution of visual attention span (VAS) remains limited. Studies in Indo-European languages have reported a direct and unique influence of VAS on reading, a relationship that might also be expected in Arabic. However, the recognition of the complex Arabic letters may require substantial attentional resources, thereby reducing the direct contribution of VAS to reading.

**Methods:**

We assessed PA, RAN, LK and VAS in Arabic-speaking beginning readers, along with their reading fluency for both nonsense syllables and real words.

**Results:**

Strong relationships were found between all four predictors and both reading outcomes. LK and VAS were also substantially related. PA and VAS were unique predictors of reading, independent of RAN. However, the direct link between VAS and reading disappeared once LK was included as an additional predictor. VAS then only contributed indirectly to reading through its influence on LK.

**Discussion:**

These findings suggest that a large share of attentional resources is required for the parallel, fine-grained processing of the multiple visual features of Arabic letters, thus taxing the attentional resources available for processing higher-order units. We therefore argue that the relationship between VAS and reading is modulated by the language script.

## Highlights

- Syllable awareness and letter knowledge (LK) are early reading predictors in Arabic.
- The visual attention span (VAS) indirectly contributes to reading in Arabic.
- This indirect contribution is fully mediated by LK, which was not observed in French.
- Multiple feature integration for letter recognition involves visual attention.
- The VAS-reading association may vary across languages depending on graphic complexity.

## 1 Introduction

The purpose of this study was to contribute to research on the cognitive skills underlying reading acquisition. Most previous studies have focused on the Indo-European languages, identifying rapid automatized naming (RAN), phonological awareness (PA) and letter knowledge (LK) as early predictors of learning to read. The present study focuses on reading acquisition in Arabic. Relatively few studies on reading predictors and reading-related skills have focused on the Arabic language, even though the specific features of this language present particular challenges. In addition to PA, RAN and LK, the present study investigates the potential contribution of visual attention span (VAS). VAS is a measure of multi-element parallel processing that reflects the amount of visual attention available for processing (Valdois, 2022). Meta-analyses suggest that VAS is an independent predictor of reading acquisition and developmental dyslexia (Liu et al., 2023; Perry and Long, 2022). While its influence on reading has been reported across several languages, evidence for its involvement in Arabic remains scarce.

### 1.1 Arabic language specific features

Arabic is a Semitic language with a rich historical and cultural heritage, distinguished by unique script and linguistic features that contribute to its complexity. Like other Semitic languages, Arabic is written from right to left along a horizontal line. It employs an ABJAD writing system wherein its fundamental script comprises consonants, with optional symbols for denoting short vowels and other morpho-phonemic features of the language (Daniels, 2013). The writing system is substantially more complex in Arabic than in languages using the Latin alphabet (Verhoeven and Perfetti, 2022). The Arabic alphabet consists of 28 letters, including two semi-vowels that can function as either a consonant or a long vowel depending on context. Written in a cursive style, Arabic orthography features connected letters with no uppercase counterparts. However, the form of Arabic letters varies based on their within-word position (initial, medial, or final). For example, the letter “Kaaf” 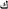 /k/ is written as 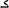 in the initial position, as 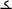 in the medial position, and as 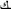 in the final position. These distinct letter forms, known as “allographs,” encompass over one hundred variations in the Arabic script. Additionally, despite the cursive nature of the Arabic script, six out of the twenty-eight letters (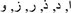 ) do not connect to the following letter, resulting in one or more spaces within words.

Furthermore, many Arabic letters share similar shapes, differing only in the presence and positioning of dots or points (known as primary diacritics), such as the letters 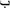 /b/, 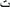 /t/, and 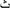 /θ/. Short vowels, in Arabic orthography, are represented by secondary diacritics above or below the letter, with three main vowels: /a/  or /fatḥa/, /u/  or /ḍamma/, and /i/  or /kasra/. The absence of a vowel is denoted by ° or /sukūn/. Certain diacritical marks, such as the /tanwīn/ (or nunation), indicating an indefinite noun through vowel doubling, and the /šadda/ (or gemination), representing consonant doubling, are considered morpho-phonemic (Saiegh-Haddad and Henkin-Roitfarb, 2014). It is important to note that vowelization in Arabic script is optional. Short vowel signs (secondary diacritics) may or may not be included, leading to two versions of the Arabic script: vowelized and unvowelized. The vowelized version is prevalent in classical Arabic texts like the Holy Qur’an, classical poems, and literacy books for young learners. The unvowelized version, devoid of short vowel markings, is used by proficient readers in books, novels, media, etc., and is introduced to children around the fourth grade to gradually familiarize them with reading in its unvowelized form. The two scripts impose different constraints on the cognitive system of reading. The vowelized script is fully transparent while the unvowelized script transcribes only part of the word phonological form (Abu-Rabia, 2001).

Another fundamental characteristic of the Arabic language is its diglossic nature, wherein it manifests in two distinct forms: the standard variety and the spoken variety. The Modern Standard Arabic (MSA) form adheres to defined rules and grammar, serving as a shared language among all Arabic speakers. It is predominantly used in written form and finds application in formal settings, religious discourse, and media communications (Versteegh, 2014). In contrast, the spoken variety is employed in everyday conversations and exhibits geographical variations across regions and even within the same country. This spoken form serves as the primary language of Arabic speakers, acquired naturally through familial interactions. Exposure to MSA typically begins during formal education, often in kindergarten. Notably, a linguistic gap exists between spoken Arabic and MSA, encompassing differences in phonology, lexicon, syntax, and morphosyntax. These disparities position MSA as a second language for young learners (Saiegh-Haddad and Schiff, 2016).

Last, Semitic morphology differs from that of European languages due to its unique non-concatenative derivational structure (Deutsch et al., 2018). Arabic morphology relies on a system of discontinuous morphemes known as roots and patterns. The root, typically composed of three consonants, indicates a semantic field, and serves as the foundation for deriving numerous words of the same semantic family. The specific meaning of each word results from the combination of the root with a pattern that corresponds to a set of vowels (and sometimes additional consonants). For instance, from the root KTB, denoting the realm of writing, arise words such as /KaTaBa/ (he wrote), KaaTiB (writer), KiTaaB (book), and maKTaBa (library). There is evidence that the morphological structure of Arabic words has an impact on reading accuracy and comprehension (Abu-Rabia, 2007; Boudelaa, 2014). Note that the impact of morphological processing on reading acquisition is beyond the scope of this paper.

### 1.2 Reading-related skills

Phonological awareness, RAN, LK and VAS have been identified as reading-related skills in Indo-European languages and as significant or potential predictors of reading in Arabic.

Rapid automatized naming requires the rapid and accurate naming of arrays of familiar items, such as letters, digits, colors, or objects. RAN is a robust predictor of reading across Indo-European languages (Araújo et al., 2015; Araújo and Faísca, 2019; Georgiou et al., 2016; Landerl et al., 2019). The predictive strength of RAN is particularly pronounced for RAN-letters or RAN-digits tasks, although RAN-colors and RAN-objects also significantly predict reading achievement in pre-readers (Lervåg and Hulme, 2009). Evidence from Arabic similarly highlights the role of RAN: it contributes to reading (Asadi et al., 2017; Hassanein et al., 2023; Ibrahim, 2015) and serves as an early predictor of reading speed, independently of PA (Asaad and Eviatar, 2014).

Phonological awareness, the ability to recognize and manipulate the phonological units of spoken language, is a key predictor of reading achievement in all languages. Strong PA skills facilitate accurate decoding and fluent reading (Duncan, 2018; Ehri et al., 2001; Melby-Lervåg et al., 2012). In Arabic as well, PA is consistently reported as a strong predictor of reading proficiency (Layes et al., 2023; Makhoul, 2017; Saiegh-Haddad and Taha, 2017; Taibah and Haynes, 2011; Tibi and Kirby, 2018). Good PA in preschool Arabic children is critical for literacy development (Mansour-Adwan et al., 2023) and PA has been shown to uniquely predict reading outcomes, independently of RAN (Asadi et al., 2017; Taibah and Haynes, 2011).

Letter knowledge (LK), defined as the ability to recognize letter shapes and associate them with their corresponding names or sounds, is one of the strongest predictors of early reading skills in Indo-European languages (Foulin, 2005). LK supports the development of decoding skills (Acha et al., 2023; Kim et al., 2010) and a LK deficit is associated with poor decoding and reduced fluency (Thompson et al., 2015). In Arabic, letter identification poses particular challenges for beginning (and even, more proficient) readers (Abdelhadi et al., 2011; Eviatar and Ibrahim, 2004). Several studies have emphasized the difficulty of recognizing and discriminating Arabic letters, suggesting that letter recognition may be more demanding on visual attention, thereby resulting in slower reading speed (Hansen, 2014; Ibrahim et al., 2002). However, to our knowledge, no study has investigated the potential unique contribution of LK to reading, beyond that of PA and RAN.

A further factor influencing reading acquisition is VAS. VAS is a measure of multi-element parallel processing in the visual modality (Valdois, 2022). It differs from the concepts of perceptual span, or visual span (Frey and Bosse, 2018), and reflects the total amount of visual attention available for the simultaneous processing of multiple elements. VAS contributes to decoding, word recognition and reading fluency, beyond PA and RAN (Bosse and Valdois, 2009; Valdois et al., 2021). It is also an early predictor of later reading skills (Valdois et al., 2019a). The few studies, that have investigated the contribution of VAS to reading in Arabic, have led to inconclusive findings. In expert readers, Awadh et al. (2016) reported no effect of VAS on reading unvowelized texts. By contrast, Lallier et al. (2018) found a relationship between VAS and text reading in Grade 4 Arabic children, but only among those with greater proficiency in reading unvowelized scripts. Last, Awadh et al. (2022) reported that VAS uniquely contributed not only to word and pseudoword reading but also to text fluency and comprehension in Grade 4–5 children. Taken together, these mixed findings underscore the need for further research into the VAS-reading relationship in the Arabic language.

Overall, RAN, PA, LK and VAS are well established as early and independent predictors of reading achievement in Indo-European languages. In Arabic, the respective roles of PA and RAN have also been investigated, with similar evidence for their unique contribution to reading. By contrast, the potential contribution of LK has yet to be examined independently of PA and RAN, and the status of VAS as an early and independent predictor of reading is still debated. Theoretical models of reading offer valuable insights into how these different skills influence reading acquisition, thereby enhancing our understanding of their unique contributions and complex interrelationships.

### 1.3 Theoretical models

According to self-teaching models (Ziegler et al., 2014; Pritchard et al., 2018), stronger PA facilitates the acquisition of decoding skills and sight-word recognition, thereby supporting more efficient reading. By contrast, the cognitive mechanisms underlying the RAN-reading relationship are still debated (Decker et al., 2013; Georgiou and Parrila, 2020). Although RAN shares many visual, attentional, language and articulatory processes with reading, no specific mechanism has been conclusively identified to account for its unique contribution to reading development.

In contrast, the contribution of letter recognition and visual attention is more clearly accounted for in computational models of reading. All reading models (Coltheart et al., 2001; McClelland and Rumelhart, 1981; Perry et al., 2007; Phénix et al., 2025) posit that word recognition is letter-based. All assume that letters are recognized through sensory information extracted from the input word. Efficient and accurate letter identification enables rapid activation of words’ orthographic representation in long term memory and its mapping onto the corresponding phonological word, thus supporting fluent reading. However, these models do not postulate that the letter itself is the basic unit of processing. Rather, letter identification is assumed to be feature-based (Grainger et al., 2008; McClelland and Rumelhart, 1981). Incoming visual information from the letter is first analyzed by detectors specialized in the processing of visual features, such as horizontal and vertical lines, intersections, curvatures or points (Dehaene et al., 2005). Letter recognition occurs through the detection and integration of these features. Some theories assume that feature binding for letter identification is achieved through visual attention (Laberge and Samuels, 1974; Treisman and Gelade, 1980).

**FIGURE 1:**
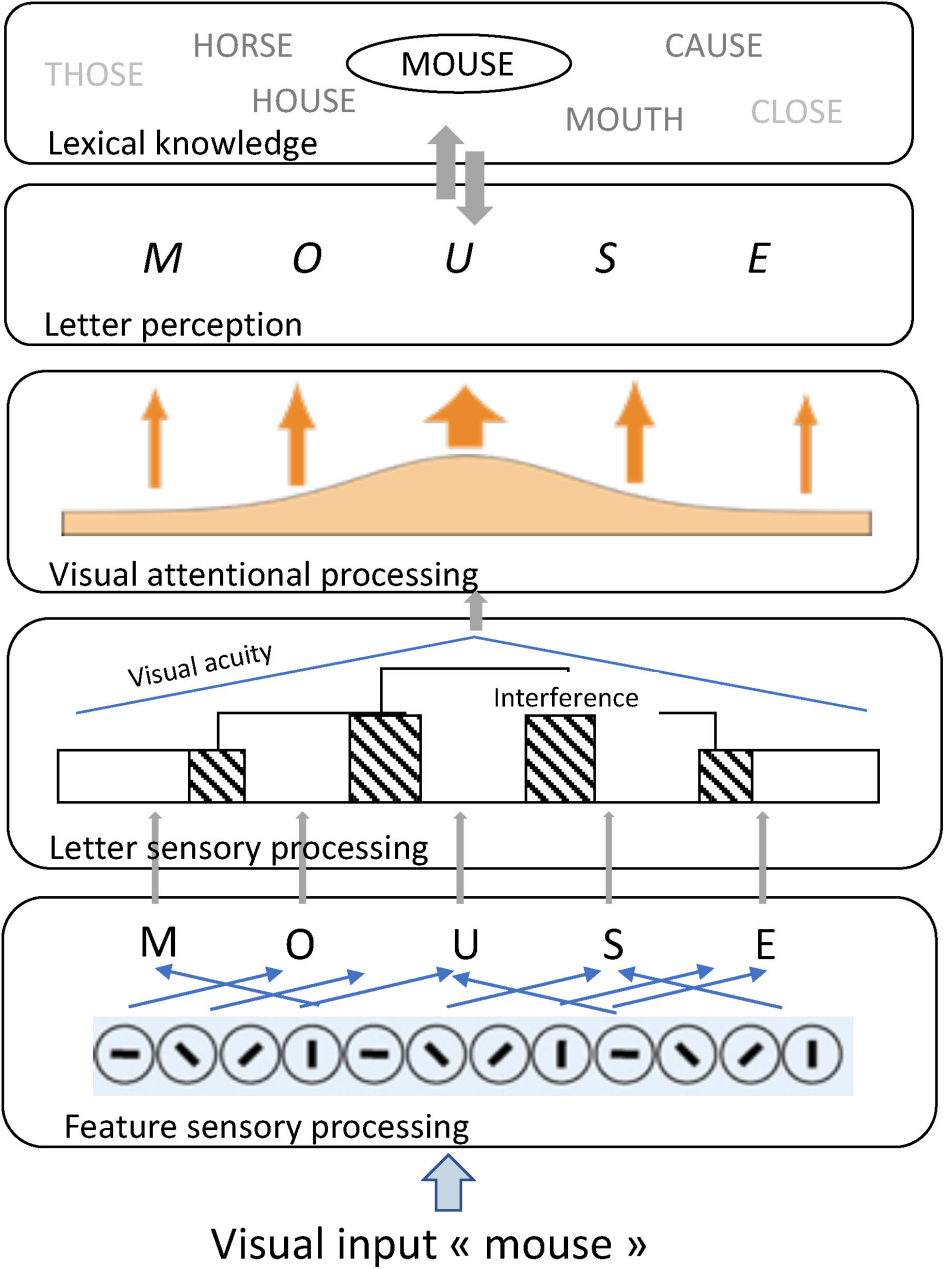
Schematic representation of the BRAID model of word recognition (Phénix et al., 2025): illustration for the input word “mouse.”

Recent models provide insights on the role of visual attention in letter and word processing (Ginestet et al., 2019, 2022; Phénix et al., 2025; Steinhilber et al., 2023). In the BRAID model of word recognition (Phénix et al., 2025), illustrated on Figure 1, visual attention intervenes between the sensory and perceptual levels of processing to promote letter recognition.

Letter recognition is challenging at the sensory level, due to both the characteristics of the alphabetic system and the properties of the visual system. Sensory processing primarily focuses on visual features, making letter identification more difficult when a letter shares numerous features with other letters in the alphabet. Additionally, letter visibility within word is degraded by the decline in visual acuity with increasing distance from fixation and by interference from adjacent letters. Visual attention has the potential to compensate for these deleterious effects, as allocating attention to letters improves their discriminability and visibility.

However, visual attention capacity (i.e., the total amount of visual attention available for processing) varies across readers. A high visual attention capacity allows simultaneous processing of multiple letters, facilitating rapid decoding and word recognition. Conversely, a lower visual attention capacity requires focusing on fewer letters for accurate recognition, resulting in more serial reading (Steinhilber et al., 2023).

This theoretical framework yields two key predictions. First, it predicts that letter processing requires more attentional resources when letters are visually complex and difficult to discriminate (i.e., sharing many features with other letters). Second, it predicts that within-word letter recognition depends on the amount of visual attention available for processing, a subject-dependent capacity that can be estimated through VAS tasks.

### 1.4 The present study

In the present study, our primary aim was to determine whether LK and VAS make unique contributions to the reading performance of Arabic beginning readers, independent of PA and RAN. While previous studies have examined the influence of some of these predictors, none have simultaneously considered all four. Oral vocabulary knowledge was also included, based on evidence that vocabulary has a positive influence on learning to read and the development of orthographic knowledge (Ziegler et al., 2014).

We began with the observation that most previous studies on reading in Arabic have used Arabic letters as stimuli to assess LK, VAS and RAN. However, RAN and VAS skills can be assessed using a variety of item types. Assuming that using the same stimuli across all three tasks would artificially strengthen the relationships among predictors and reduce their potential unique contribution to reading, we opted to use different items for assessing LK, RAN and VAS. LK is necessarily evaluated using the letters of the alphabet, whereas RAN can be measured using either alphanumeric or non-alphanumeric items to tap rapid access to phonological labels, fast visuo-verbal matching and/or processing speed. Participants were administered a RAN-objects task, based on prior evidence for a relationship between this version of the task and reading in Arabic (Layes et al., 2017). VAS is typically assessed through the oral report of alphanumeric stimuli to estimate the amount of visual attention allocated to the simultaneous processing of multiple visual elements (Valdois, 2022). Leveraging participants’ knowledge of English, the VAS report tasks were administered using Latin letters. This approach – using Arabic letters for the assessment of LK, objects for RAN and Latin letters for VAS – ensured that each fundamental skill was specifically targeted.

Consistent with previous behavioral evidence and current models of self-teaching (Ziegler et al., 2014; Pritchard et al., 2018), higher PA at the onset of literacy instruction should facilitate the development of decoding skills and thus predict higher performance in reading nonsense syllables and words in vowelized script. As previously reported, RAN should account for a significant proportion of variance in Arabic reading, over and above PA.

Theoretical models of visual attention posit that visual attention is involved in feature binding for letter recognition and in parallel multiletter processing for letter-chunk and word recognition. Given the particular challenge of Arabic letter identification for beginning readers, we predict a contribution of VAS to letter recognition (LK). According to the BRAID model, visual attention further facilitates letter-chunk and word recognition by compensating for the detrimental effects of visual acuity decline and crowding on letter identification within strings. Therefore, we predict a relationship between VAS and reading fluency.

However, visual attention is a capacity-limited resource. When greater attentional resources are required for letter feature identification, fewer resources remain available for letter-chunk and word recognition processing. Therefore, we expect VAS to exert either a direct residual effect on reading after controlling for LK, or only an indirect effect on reading, in case of full mediation by LK.

Reading skills were assessed through tasks of nonsense syllable (viewed as legal monosyllabic pseudo-words) and word reading. Evidence from Indo-European languages suggests that PA, RAN, LK and VAS contribute differently to word and pseudo-word reading (Georgiou et al., 2008; Shapiro et al., 2013). Accordingly, we will examine the predictive power of these different skills separately for each reading measure.

## 2 Materials and methods

### 2.1 Participants

One hundred and thirty-four bilingual Lebanese first graders were recruited from four private schools with mild to low socio-economic levels. All children had normal hearing and either normal or corrected-to-normal vision. They had completed 3 years of kindergarten and began formal reading instruction in Grade 1. Their native language was Lebanese Arabic. From kindergarten onward, children were exposed to both Arabic and English. Oral instruction in Arabic consisted in a combination of Modern Standard Arabic (MSA) and Lebanese Arabic, adapted to the children’s comprehension and oral expression abilities. Written instruction in Grade 1 was exclusively in MSA. English reading instruction also began in Grade 1, and both Arabic and Latin letters had been formally introduced in KG3. Testing occurred after the second trimester of the academic year, after 4 months of formal literacy instruction.

The study was conducted in accordance with the ethical principles outlined in the Declaration of Helsinki. Ethical approval for the study (PASEM Project: E.T. as PI) was granted by the Ethics Committee of the Africa Institute for Research in Economics and Social Sciences (under grant: ECAIRESS-002-2024). Legal responsibility for the children during school hours was assumed by the school principals, who consented to the assessments. Written informed consent was obtained from all parents for their child’s participation. Additionally, verbal assent was secured from each child at the beginning of each testing session, with reassurance that they could withdraw at any time.

Due to absences during at least one data collection session, complete data were not available for all children: 18 data points were missing for reading tasks, 23 for VAS tasks and 10 for PA tasks, resulting in the exclusion of 32 children (24%). In addition, we excluded one extreme outlier, likely due to a measurement error during the reading task (120 syllables correctly read per minute). The final sample comprised 101 first graders (58 females) with a mean age of 6 years and 11 months (SD = 4.1 months).

### 2.2 Measures

Most of the tasks administered in this study were custom-designed due to the absence of standardized assessment for beginning Arabic readers.

#### 2.2.1 Non-verbal reasoning test (Raven)

Abstract reasoning was assessed using Raven’s Colored Progressive Matrices (RCPM) as a measure of fluid intelligence (Raven, 2000). Participants were presented with three series of 12 colored matrices, each with a missing element. For each item, they were asked to select the missing element from a set of options provided below the matrix. Given the absence of normative data for the Lebanese population, raw scores were used for analysis (maximum score = 36).

#### 2.2.2 Vocabulary knowledge (Voc)

Oral vocabulary was assessed using the object naming subtest of the ELO-L, a language screening tool for Lebanese children aged 3–8 years (Zebib et al., 2019). The test consisted of 35 pictures presented individually. Participants were encouraged to respond in standard Arabic but answers in Lebanese Arabic were accepted when they were unfamiliar with the standard Arabic label. The score was calculated as the total number of correct responses, irrespective of the language register (maximum score = 35).

#### 2.2.3 Rapid automatized naming (RAN)

Rapid automatized naming was assessed using a custom-designed RAN-objects task, requiring the participants to name a series of familiar objects as quickly as possible. The task consisted of 5 different objects, each repeated 5 times in random order. All words were monosyllabic, high frequency words, according to the ALEF frequency database (Abou Melhem and Badran, 2022), and showed minimal variation between MSA and spoken Lebanese Arabic: bear (/dub/ in MSA - /dib/ in Lebanese), rooster (/diik/-/diik/), hand (/yad/-/iid/), elephant (/fiil/-/fiil/), and house (/bayt/-/beet/). All 25 pictures were presented in rows to be scanned from right to left. The experimenter recorded the total completion time, expressed in seconds.

#### 2.2.4 Phonological awareness (PA)

Phonological awareness was assessed using three tasks of Syllable Segmentation (SylSeg), Initial Syllable Deletion (SylDel), and Initial Phoneme Deletion (PhonDel). The Syllable Segmentation task was specifically designed for this study, whereas the Syllable and Phoneme deletion tasks were from the BELEA battery (Henry et al., in press). Following the recommendations of Saiegh-Haddad et al. (2020), all tasks were administered in MSA, using items that minimally differed from spoken Lebanese Arabic. Each task comprised 8 items, preceded by practice trials. Scores were expressed as the number of correct responses per minute.

In the Syllable Segmentation task, participants had to segment 5 bisyllabic words with simple CV and CVV syllables (e.g., /saa-’a/ meaning “hour” or “clock”) and 3 trisyllabic words containing, at least, one complex CVC syllable (e.g., /laa-’i-bun/ meaning “player”).

In the Syllable Deletion task, children were instructed to delete the first syllable of familiar 2- or 3-syllable words and produce the remaining sequence. The deleted syllables were either simple CV and CVV syllables (e.g., /baa-ri-dun/ meaning “cold”) or complex CVC syllables (e.g., /Sham-sun/ meaning “sun”).

In the Phoneme Deletion task, participants had to remove the initial phoneme from words consisting of 2-to-3 syllables and 5-to-7 phonemes. To reduce memory load, all words shared their last 2 phonemes, corresponding to the nunation /un/ indicating that they were syntactically indefinite. In 5 of the 8 items, the deleted phoneme occurred in a long syllable where the vowel was represented by a full letter (e.g., /fii-lun/ meaning “elephant”), and in the remaining 3 items, it occurred in a short syllable where the vowel was marked by a diacritic (e.g., /qal-bun/ meaning “heart”).

#### 2.2.5 Letter knowledge (LK)

Letter knowledge was evaluated through 3 tasks of Arabic letter naming, allograph naming and allograph designation.

In the Arabic Letter Naming (LetName) task, the 28 Arabic letters were presented individually in a random order. Children were instructed to name each letter as quickly and accurately as possible. They were asked to produce the name of the letter; however, some participants responded with the letter sound combined with the vowel /a/ (e.g., /da/ for the letter /daal/; /sa/ for the letter /siin/). Following previous studies (Tibi et al., 2021), both response types were scored as correct (maximum score = 28).

In the Allograph Naming (AlloName) task, participants were asked to name 28 isolated allographs presented individually. As for letter naming, both the letter name or the letter sound were accepted as correct responses. The score corresponded to the total number of correct responses (maximum score = 28).

In the Allograph designation (AlloDes) task, each trial consisted of 4 allographs corresponding to different letters. The examiner provided the name of the target letter, and the child was instructed to identify the corresponding allograph. The score was the number of correct responses (maximum score = 28).

#### 2.2.6 Visual attention span (VAS) and single letter identification threshold (SLIT)

Visual attention span was assessed using global and partial letter report tasks (Valdois, 2022). While strings of 5 letters are typically used for VAS assessment in primary school children from Indo-European languages, previous studies have shown that 5-letter strings in Arabic is too difficult, even for advanced readers (Awadh et al., 2016, 2022). A pilot study confirmed that 4-letter strings were appropriate for beginning readers of Arabic.

The 4-letter strings (e.g., R H S D) were constructed from ten Latin consonants (B, P, T, F, L, M, D, S, R, H). Letters were presented in uppercase (Arial Font, 7 mm high) in black on a white background, without repeated letters or real-word patterns. Each string subtended an angle of 4.2° at a 50 cm viewing distance. Inter-letter spacing was increased to 0.57° (edge-to-edge), to minimize lateral interference. Tasks were administered using the E-Prime software.

Each trial began with a central fixation point displayed for 1,000 ms, immediately followed by a blank screen for 500 ms. The 4-letter string was then presented for 200 ms. It was centered on the fixation point to ensure equal and optimal acuity for all letters, regardless of their position in the string. In the global report task, 20 4-letter strings were presented successively. Participants had to report as many letters as possible in any order, at the string offset. In the partial report task, each briefly presented 4-letter string was immediately followed by a cue indicating the position of the letter to be reported. Forty trials were administered (10 targets per position). In both conditions, feedback was provided during training but withheld during experimental trials. One point was awarded for each correct letter, with a maximum score of 80 in global report and 40 in partial report.

A single-letter identification threshold task was also administered to control for individual differences in processing single Latin letter. The 10 consonants used in the report tasks were presented individually for durations randomly varying between 33 ms and 101 ms (in 16 ms increments), followed by a mask to erase information in iconic memory. One point was awarded for each accurately named letter. Following Bosse and Valdois (2009), a weighted score was computed: 5 × score at 33 ms + 4 × score at 50 ms + 3 × score at 67 ms, 2 × score at 84 ms, + 1 × score at 101 ms, for a maximal score of 150.

#### 2.2.7 Reading

Reading fluency was assessed using three custom-made lists of 12 items each, targeting nonsense syllables, monosyllabic words, and polysyllabic words. All items were written in a fully transparent, vowelized script and consisted of 2-to-4 letter strings, chosen to minimize discrepancies between spoken Lebanese and Standard Arabic. Items were listed in columns, and each list was read separately. The list of nonsense syllables (from 1 to 2 letters) included 5 short syllables (CV, e.g., /si/ 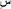), with varying consonant frequencies and no rare consonants (Boudelaa et al., 2020), and 7 long syllables (CVV, e.g., /fuu/ 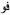), characterized by a high level of discriminability for five of them and a high level of difficulty for the remaining two (Tibi et al., 2021).

The monosyllabic word list consisted of complex words (2-to-3 letter long), including frequent patterns according to the ALEF database (Abou Melhem and Badran, 2022). They followed either a CVC (e.g., /Ɂaχ/ meaning “brother”), CVVC (e.g., /ҁiid/ meaning “holiday”), or CVCC (e.g., /Ṣayf/ meaning “summer”) structure.

The polysyllabic word list contained simple words of 2-to-3 syllables (3-to-4 letter long), drawn from frequent CVV-CV (e.g., /naa-ma/ meaning “he slept”) or CV-CV-CV (e.g., /ka-ta-ba/ meaning “he wrote”) patterns. Participants were instructed to read each list as quickly and accurately as possible. Reading fluency was scored as the number of accurately read items per minute.

### 2.3 Data collection and analysis plan

Data collection was conducted by trained experimenters, all of whom had background in speech and language therapy. The tests were administered individually in a quiet room at school. To control for fatigue effects, the order of task administration was randomized across participants. Each child completed the assessment in 2-to-3 sessions, each lasting approximately 35–50 min. Following data collection, scoring was performed by the experimenters and cross-verified twice to ensure accuracy. Reliability estimates for the different measures were calculated using McDonald’s Omega coefficients (Hayes and Coutts, 2020) and are reported in Table 1.

**TABLE 1:** Mean (M), standard deviation (SD), median (Mdn), minimum (Min.), maximum (Max.), skewness (Skew.), kurtosis (Kurt.), data transformation applied in further analysis (Transf.) and McDonald’s Omega reliability coefficient (ω) for all variables.

Variable	*M*	SD	Mdn	Min	Max	Skew.	Kurt.	Transf.	ω
Raven	17.18	4.24	17.00	6.00	31.00	0.48	1.18		0.76
Vocabulary	12.37	5.38	13.00	1.00	25.00	0.07	−0.66		0.85
RAN	39.39	17.93	34.00	19.00	118.00	2.20	5.51	1/*x*	NA
LK	58.39	16.01	62.00	19.00	82.00	−0.56	−0.67		0.96
LetName	20.19	5.82	22.00	6.00	28.00	−0.64	−0.52		0.91
AllogName	16.84	6.21	17.00	3.00	28.00	−0.25	−0.84		0.91
AllogDes	21.36	4.89	23.00	9.00	28.00	−0.72	−0.36		0.87
PA	5.34	3.27	4.82	0.35	16.98	1.17	1.42	$\sqrt{x}$	0.85
SylSeg	9.57	4.80	9.06	0.44	20.87	0.20	−0.63		0.84
SylDel	3.86	3.28	3.00	0.00	15.56	1.26	1.25		0.85
SLIT (Latin)	90.04	34.58	96.00	6.00	150.00	−0.22	−1.01		0.96
VAS	57.28	16.38	56.25	17.50	89.38	−0.09	−0.50		0.88
Partial report	24.89	8.20	25.00	3.00	38.00	−0.49	−0.35		0.83
Global report	41.86	12.63	40.00	12.00	73.00	0.33	−0.40		0.83
Syllable fluency	11.33	13.60	6.00	0.00	80.00	2.30	6.60	$\sqrt{x}$	0.91
Word fluency	4.45	5.76	2.75	0.00	28.80	2.31	5.56	$\sqrt{x}$	0.95
Monosyllables	3.75	5.39	2.09	0.00	24.44	2.57	6.39		0.89
Multisyllables	5.67	7.06	2.50	0.00	36.00	2.07	4.61		0.92

LetName = letter naming; AllogName = allograph naming; AllogDes = allograph designation; SylSeg, syllable segmentation; SylDel, syllable deletion; SLIT Latin, single letter identification threshold for Latin letters; Syllable and word fluency, number of syllables/words accurately read per minute. RAN, rapid automatized naming; LK, letter knowledge; PA, phonological awareness; VAS, visual attention span.

For statistical analyses, we first computed correlation matrices, which revealed a strong association between two potential predictors of reading fluency: Visual attention span and Letter knowledge (LK). We then conducted two sets of regression analyses: one excluding Letter knowledge and one including it as a predictor. Finally, structural equation modeling (SEM) was employed to further examine the specific contributions of these two correlated predictors to reading fluency.

## 3 Results

### 3.1 Descriptive statistics

A substantial floor effect was observed on the Phoneme Deletion task, with 68% of children scoring 0 out of 8. Consequently, this measure was excluded from further analysis. Table 1 reports descriptive statistics for all remaining variables, as well as for the four composite measures of PA, LK, VAS and Word Reading. The PA composite score was theoretically justified and calculated by averaging performance on the Syllable Segmentation and Syllable Deletion tasks. The LK composite score was obtained by summing performance across the three tasks of Letter Naming, Allograph Naming and Allograph Designation. The Word Reading composite score corresponded to the average performance on the mono- and poly-syllabic words. To balance the contributions of the global and partial report tasks, the VAS composite score was calculated using the following formula:


$$T\,S_{V\,A\,S}=\frac{\left(G\,l\,o\,b\,a\,l_{s\,c\,o\,r\,e}+\;2\times P\,a\,r\,t\,i\,a\,l_{s\,c\,o\,r\,e}\right)\times 100}{80\;+\;\left(2\times 40\right)}$$

As shown in Table 1, McDonald’s Omega coefficients for all variables, including the four composite constructs, ranged from 0.83 to 0.96 (with the exception of the Raven test: ω = 0.76), indicating good internal consistency of the measurement instruments.

The distributions of most variables (Raven, Vocabulary, LK, SLIT and VAS) approximated normality, with skewness values ranging from −0.56 to +0.48 and kurtosis values from −1.01 to 1.18. The distribution was moderately skewed for PA and highly skewed and peaked for the RAN, Syllable Fluency and Word Fluency measures. To increase symmetry and reduce kurtosis, square root transformations were applied to all variables except RAN, which underwent an inverse transformation. After transformation, skewness and kurtosis values fell within acceptable ranges (skewness: −0.56 to 1.01; kurtosis: −1.01 to 1.18; see Supplementary Table 1).

Before computing composite scores of LK, VAS and Word Reading, we verified that the component scores for each construct were significantly correlated (see Supplementary Table 2). Strong correlations were observed among the three LK tasks (from *r* = 0.82 to 0.87, all p_*s*_ > 0.001). Significant associations were also found between Partial and Global Report tasks [*r*(99) = 0.59, *p* < 0.001], and between monosyllabic and polysyllabic words [*r*(99) = 0.86, *p* < 0.001]. Furthermore, high internal consistency for aggregated items (ω_*PA*_ = 0.85; ω_*LK*_ = 0.96; ω_*VAS*_ = 0.88; ω_*Word Reading*_ = 0.95) justified the use of composite scores in subsequent analyses.

### 3.2 Correlation analyses

Table 2 presents the zero-order and partial correlation coefficients among all main variables. Partial correlations were computed while controlling for RAVEN, to ensure that the observed associations between variables were not solely attributable to general cognitive skills.

**TABLE 2:** Pearson correlations (above the diagonal) and partial correlations (below the diagonal) after control of Raven.

	Vocab.	RAN	LK	PA	SLIT	VAS	ReadSyll	ReadWords
Raven	0.14	0.25	0.21	0.27	0.15	0.38**	0.33	0.31
Vocab.	–	0.28	0.22	0.01	0.12	0.09	0.19	0.19
RAN	0.25	–	0.19	0.32	0.06	0.26	0.35*	0.35*
LK	0.19	0.15	–	0.33*	0.28	0.59***	0.79***	0.74***
PA	−0.03	0.27	0.29	–	0.31	0.43***	0.50***	0.58***
SLIT	0.11	0.03	0.26	0.28	–	0.40**	0.29	0.25
VAS	0.04	0.18	0.57***	0.37*	0.37**	–	0.53***	0.58***
ReadSyll	0.16	0.29	0.78***	0.45***	0.26	0.46***	–	0.84***
ReadWords	0.15	0.30	0.72***	0.54***	0.22	0.52***	0.82***	–

*p < 0.05;

**p < 0.01;

***p < 0.001; *p*-values are adjusted using Bonferroni correction for 64 tests. Vocab., Vocabulary; RAN, Rapid Automatized Naming; LK, Letter knowledge; PA, Phonological Awareness; SLIT, Single Letter Identification Threshold; VAS, Visual Attention Sapn; ReadSyll, Syllable Reading; ReadWords, Word Reading.

As shown on Table 2, the predictive variables of PA, LK and VAS were significantly correlated with the two dependent measures of nonsense syllable and word reading. RAN was significantly correlated with these two reading measures, but only when Raven’s scores were not controlled for. In contrast, Vocabulary did not show significant associations with any variable and was therefore excluded from subsequent predictive analyses. The strongest correlations were observed between the two (word and syllable) reading measures and LK. However, significant intercorrelations were also found among some predictors. In particular, VAS correlated moderately with LK [*r*(99) = 0.57, *p* < 0.001] and, to a lesser extent, with PA [*r*(99) = 0.37, *p* < 0.05]. VAS was also correlated with the control variable of single-letter identification threshold (SLIT).

### 3.3 Regression analyses and structural equation modeling

Simple regression analyses were first performed with either Syllable Reading or Word Reading as dependent variables, and PA, RAN, VAS, Raven, and SLIT as independent variables. The analyses (see Supplementary Tables 3a, b) revealed that the contribution of RAN did not significantly predict Word Reading fluency (*t* = 1.56, *p* = 0.12, *R_*p*_^2^* = 0.03), and its effect on Syllable Reading fluency was only marginally significant (*t* = 1.83, *p* = 0.07, *R_*p*_^2^* = 0.03). Only PA and VAS emerged as significant predictors of reading skills (PA: *t* = 2.99, *p* = 0.004, *R_*p*_^2^* = 0.09, and *t* = 4.41, *p* < 0.001, *R_*p*_^2^* = 0.17; VAS: *t* = 3.21, *p* = 0.002, *R_*p*_^2^* = 0.10, and *t* = 4.19, *p* < 0.001, *R_*p*_^2^* = 0.16, for Syllable and Word Reading, respectively).

However, when LK was introduced as an additional predictor, PA remained a significant predictor of both syllable and word reading fluency (Syllables: *t* = 3.37, *p* = 0.001, *R_*p*_^2^* = 0.11; Words: *t* = 4.92, *p* < 0.001, *R_*p*_^2^* = 0.20), whereas the predictive contribution of VAS disappeared (Syllables: *t* = −0.95, *p* = 0.34, *R_*p*_^2^* = 0.01; Words: *t* = 0.83, *p* = 0.41, *R_*p*_^2^* = 0.01). A significant effect of RAN was found, but only for Syllable Reading (Syllables: *t* = 2.28, *p* = 0.03, *R_*p*_^2^* = 0.05; Words: *t* = 1.72, *p* = 0.09, *R_*p*_^2^* = 0.03). Notably, LK emerged as the strongest predictor for both Syllable Reading (*t* = 10.37, *p* < 0.001, *R_*p*_^2^* = 0.53) and Word Reading (*t* = 7.98, *p* < 0.001, *R_*p*_^2^* = 0.40). Together with the substantial correlation between LK and VAS (*r* = 0.57), these findings suggest that the effect of VAS on reading performance is likely indirect, mediated through LK.

**FIGURE 2:**
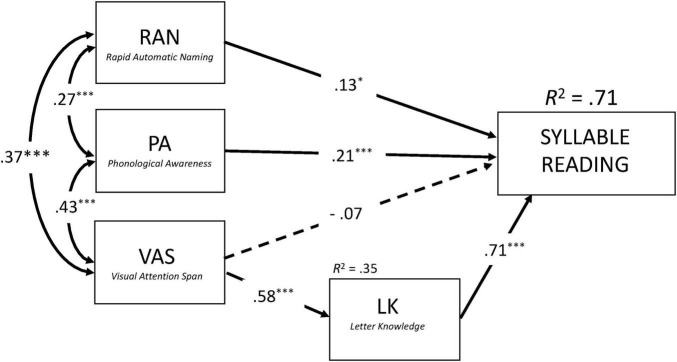
Concurrent predictors of syllable reading after controlling for Raven and single letter identification threshold (SLIT). Results from the structural equation model (SEM) with standardized coefficients. Bi-directional arrows are for covariance and uni-directional arrows for regression coefficients. Solid arrows and dashed arrows represent significant and non-significant relations, respectively. *p < 0.05; ***p < 0.001.

**FIGURE 3:**
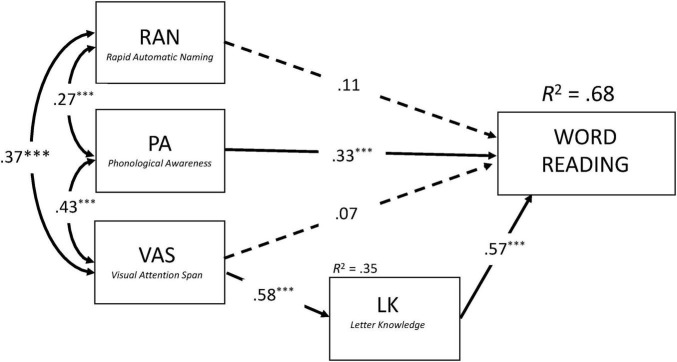
Concurrent predictors of word reading after controlling for Raven and single letter identification threshold (SLIT). Results from the structural equation model (SEM) with standardized coefficients. Double arrows correspond to covariance. Simple arrows correspond to regression coefficients. Solid arrows and dashed arrows represent significant and non-significant relations, respectively. ***p < 0.001.

To directly assess this hypothesis, we performed two structural equation models while controlling for RAVEN and Single (Latin) letter identification threshold (SLIT). These models were conceptually similar to previous regression analyses, but included both the direct and indirect (through LK) effects of VAS on reading performance. As shown in Figures 2, 3, the models accounted for 71% and 68% of variance in Syllable Reading and Word Reading, respectively (see Supplementary Tables 4a, b for detailed information about the two structural models). Consistent with our predictions, VAS exerted no significant direct effect on reading fluency (Syllables: b* = −0.07, *p* = 0.33, *z* = −0.98; Words: b* = 0.07, *p* = 0.36, *z* = 0.91). Instead, its contribution was entirely indirect through LK (Syllables: b* = 0.41, *p* < 0.001, *z* = 5.39; Words: b* = 0.33, *p* < 0.001, *z* = 5.46). VAS explained 33% of the variance in LK, which in turn accounted for 50% and 32% of the variance in Syllable and Word Reading, respectively.

## 4 Discussion

The primary aim of this study was to advance our understanding of the cognitive processes involved in learning to read in beginning Arabic-speaking readers. A key strength of this work lies in its simultaneous examination of a broader set of potential predictors than typically considered. Beyond PA and RAN, we also investigated the influences of LK and VAS, with particular attention to the interrelationships between these latter skills and concurrent reading outcomes.

Consistent with self-teaching theoretical models (Share, 1999; Ziegler et al., 2014; Pritchard et al., 2018), our findings indicate that PA is a unique concurrent predictor of reading outcomes. This corroborates the widely documented association between PA and learning to read in both Indo-European languages and other language families, including Arabic (Awadh et al., 2022; Ibrahim et al., 2007; Layes and Bouakkaz, 2022; Taibah and Haynes, 2011; Tibi and Kirby, 2018). The present findings extend this body of evidence by showing that the influence of PA persists even after controlling for RAN, LK and VAS.

Although research in Indo-European languages has mainly emphasized the relationship between phoneme awareness and reading acquisition (Melby-Lervåg et al., 2012), our Arabic-speaking participants demonstrated very limited ability to identify and manipulate phonemes, although they had developed syllable awareness skills, children with stronger syllable awareness achieving better reading outcomes. It is widely acknowledged that syllable awareness develops prior to phoneme awareness (Ziegler and Goswami, 2005). Although this developmental sequence is found in all languages, cross-linguistic variations exist. Higher levels of syllable awareness are typically observed earlier in transparent orthographies, and phoneme awareness is acquired more rapidly in these languages, especially when they have simple syllabic structures (Míguez-Álvarez et al., 2022; Ziegler and Goswami, 2005). Our findings suggest that the syllable is a more accessible phonological unit than the phoneme for Arabic beginning readers. They further suggest that the acquisition of phoneme awareness may be delayed in Arabic compared to Indo-European languages. This delay in phoneme awareness acquisition could stem from the ambiguous status of the Arabic writing system, which can be described as alphabetic –considering that each written character (letters and diacritics) corresponds to a phoneme– or syllabic, if diacritics are considered as integral parts of consonant letters (Taouk and Coltheart, 2004). Finally, RAN showed only a marginal association with reading skills, may be due to the use of non-alphanumeric stimuli, which precludes any solid interpretation of the current results.

Another important finding of this study is that knowledge of Arabic letters (LK) was a strong predictor of reading performance in Arabic-speaking beginning readers. As previously noted, letter knowledge is consistently regarded as one of the most powerful –if not the most powerful– predictors of early interindividual differences in reading acquisition across languages (Foulin, 2005). Knowledge of the graphs used in a given orthographic system is universally acknowledged as an essential prerequisite for learning to read (Verhoeven and Perfetti, 2022). This holds true for Arabic as well (Asaad and Eviatar, 2014). In a longitudinal study within the framework of the Morocco Literacy Project, Wagner and Spratt (1993) examined the longitudinal effects of early letter knowledge on reading development over a 5-year follow-up. They concluded that letter knowledge was among the strongest predictors of reading achievement in Arabic.

Other studies have highlighted the challenges posed by the visual complexity and confusability of Arabic letters for learning to read. For example, Dai et al. (2013) reported that reading speed was negatively affected by the diacritical complexity of consonants. Words containing letters with multiple diacritic dots were read more slowly, suggesting that the visual complexity of Arabic letters has a direct impact on reading.

Overall, these findings suggest that phoneme awareness and letter knowledge are the two building blocks of learning to read in Arabic. The crucial role of these two skills across languages has recently been emphasized by the “Universal Combinatorial (UC) Model” proposed by Share (2025). According to this model, the initial sub-morphemic phase of learning to read relies on mastery of the basic units of spoken and written language, followed by the progressive development of higher-order orthographic units, and the establishment of orthography-to-phonology mappings. At the visual level, this developmental trajectory involves encoding increasingly larger and more complex units, through the combination of basic visual features into letters, and letters into larger chunks, like syllables, morphemes or words. However, the UC model leaves open the question of the cognitive mechanism, or the “glue,” that enables the integration of these basic units into higher-order units.

The primary contribution of our study lies in highlighting the role of visual attention, as a mechanism involved in feature integration for letter identification and, more broadly, in the parallel processing of multiple elements for the identification of larger units. Our investigation was initially motivated by prior evidence indicating that visual attention, estimated through VAS tasks, constitutes a robust concurrent and longitudinal predictor of reading across languages (Perry and Long, 2022; Liu et al., 2023). Thus, despite inconsistencies reported in previous studies (Awadh et al., 2016, 2022; Lallier et al., 2018), we expected VAS to significantly contribute to reading acquisition in Arabic.

Our results partially support this hypothesis. As anticipated, we found that VAS was a reading-related skill and that the VAS-reading relationship was independent of PA and RAN. However, the direct link between VAS and reading disappeared when LK was introduced as an additional predictor. We then observed that visual attentional resources (estimated through VAS) contributed indirectly to reading through their influence on letter recognition. In other words, beginning readers with higher visual attentional resources demonstrated more efficient Arabic letter recognition, which in turn enhanced their reading performance. This indirect effect of VAS at the beginning of literacy instruction in Arabic contrasts with previous evidence for a direct contribution in French. For instance, Valdois et al. (2019a) reported a direct and significant contribution of early VAS (assessed through digit report tasks) to later reading in French-speaking children, even after controlling for PA and LK. This suggests that the indirect contribution of visual attention to reading may be specific to Arabic, or more generally, to writing systems using complex graphs. Further cross-linguistic studies are required to determine whether the contribution of VAS varies as a function of orthographic complexity.

There is broad consensus that letter processing is feature-based (Grainger et al., 2008; Pelli et al., 2006). Behavioral (Aljassmi and Perea, 2024) and electrophysiological evidence (Carreiras et al., 2013) both support a similar and early step of feature-detection for the identification of both Latin and Arabic letters. There is also ample evidence that visual attention is required to bind features together and facilitate the discrimination of visually similar letters (Carrasco, 2011; Treisman and Gelade, 1980; Wolfe, 2003). However, because visual attention is a capacity-limited process, allocating attentional resources to one level of visual processing reduces the resources available for others. Thus, the indirect effect of visual attention on reading that we have reported in beginning readers of Arabic may reflect the higher attentional demands of letter recognition in this language, compared to those using Latin letters. Assuming that visual attention enhances feature identification and feature binding for letter recognition across languages, then Arabic letters may require more attention for the parallel and fine-grained processing of their multiple features. Consequently, most attentional resources would be devoted to the simultaneous processing of features for Arabic letter recognition, leaving fewer resources available for the parallel processing of multiple letters, and reading enhancement. In contrast, easier Latin letter recognition likely reduces the attentional cost, freeing resources for the parallel processing of letters and their integration into larger orthographic units for more fluent reading.

Currently, there is no direct evidence in support of this hypothesis, although some behavioral and neurobiological findings point in the expected direction. First insights come from a single case study of patient, IG, who suffered bilateral lesions of the superior parietal lobules (SPLs; Valdois et al., 2019b). The SPLs have been identified as the neural correlates of VAS in both typical and dyslexic readers (Lobier et al., 2012, 2014; Peyrin et al., 2011) and IG exhibited a severe VAS deficit. Investigation of her visual attentional field revealed a specific impairment for visual search involving objects constituted of separable features, compared to filled-object conditions (Khan et al., 2016). This pattern was interpreted as evidence that visual attention is involved in the integration of separable features within objects, including letters. Additional insights come from studies comparing Arabic and Latin letter writing in French-Arabic bi-scripters engaged during a copying task (Fabiani et al., 2023). The analysis revealed that Arabic letters took longer to write than Latin letters, consistent with the greater complexity of Arabic letter shapes. Interestingly, investigation of the neural networks underlying writing in the two scripts showed stronger SPL recruitment for Arabic letters, compared to Latin letters. Assuming that handwritten copying relies on detailed visual analysis of letter shapes (Seyll et al., 2020), these findings suggest that visual attention is more strongly involved in processing Arabic than Latin letters. According to this assumption, at the beginning of literacy acquisition, visual attention should influence Arabic reading indirectly through LK mediation, while a more direct contribution may gradually emerge as letter recognition becomes automatized. Future research is needed to assess this prediction.

Last, the current findings may also suggest that beginning readers of Arabic primarily adopt a letter-by-letter strategy for both syllable and word reading, a plausible consequence of allocating most visual attentional resources to letter processing, at the expense of higher-order orthographic units.

## Data Availability

The raw data supporting the conclusions of this article will be made available by the authors, without undue reservation.

## References

[B1] Abdelhadi S. Ibrahim R. Eviatar Z. (2011). Perceptual load in the reading of Arabic: Effects of orthographic visual complexity on detection. *Writ. Syst. Res.* 3 117–127. 10.1093/wsr/wsr014

[B2] Abou Melhem N. Badran D. (2022). *ALEF: arabic LExicon frequency [Database].* Lebanon: Saint-Joseph University of Beirut.

[B3] Abu-Rabia S. (2001). The role of vowels in reading Semitic scripts: Data from Arabic and Hebrew. *Read. Writ.* 14 39–59. 10.1023/A:1008147606320

[B4] Abu-Rabia S. (2007). The role of morphology and short vowelization in reading Arabic among normal and dyslexic readers in grades 3, 6, 9, and 12. *J. Psycholinguist Res.* 36 89–106. 10.1007/s10936-006-9035-6 17109241

[B5] Acha J. Rodriguez N. Perea M. (2023). The role of letter knowledge acquisition ability on children’s decoding and word identification: Evidence from an artificial orthography. *J. Res. Reading* 46 358–375. 10.1111/1467-9817.12432

[B6] Aljassmi M. A. Perea M. (2024). Visual similarity effects in the identification of Arabic letters: evidence with masked priming. *Language and Cognition* 16, 1618–1638. 10.1017/langcog.2024.20

[B7] Araújo S. Faísca L. (2019). A meta-analytic review of naming-speed deficits in developmental dyslexia. *Sci. Stud. Read.* 23 349–368. 10.1080/10888438.2019.1572758

[B8] Araújo S. Reis A. Petersson K. M. Faísca L. (2015). Rapid automatized naming and reading performance: A meta-analysis. *J. Educ. Psychol.* 107 868–883. 10.1037/edu0000006

[B9] Asaad H. Eviatar Z. (2014). Learning to read in Arabic: The long and winding road. *Read. Writ.* 27 649–664. 10.1007/s11145-013-9469-9

[B10] Asadi I. A. Khateb A. Ibrahim R. Taha H. (2017). How do different cognitive and linguistic variables contribute to reading in Arabic? A cross-sectional study from first to sixth grade. *Read. Writ.* 30 1835–1867. 10.1007/s11145-017-9755-z

[B11] Awadh F. H. Phénix T. Antzaka A. Lallier M. Carreiras M. Valdois S. (2016). Cross-language modulation of visual attention span: An Arabic-French-Spanish comparison in skilled adult readers. *Front. Psychol.* 7:178306. 10.3389/fpsyg.2016.00307 27014125 PMC4779959

[B12] Awadh F. H. Zoubrinetzky R. Zaher A. Valdois S. (2022). Visual attention span as a predictor of reading fluency and reading comprehension in Arabic. *Front. Psychol.* 13:868530. 10.3389/fpsyg.2022.868530 36483706 PMC9723150

[B13] Bosse M. L. Valdois S. (2009). Influence of the visual attention span on child reading performance: A cross-sectional study. *J. Res. Readi.* 32 230–253. 10.1111/j.1467-9817.2008.01387.x

[B14] Boudelaa S. (2014). “Is the Arabic mental lexicon morpheme-based or stem-based? Implications for spoken and written word recognition,” in *Handbook of Arabic literacy: Insights and perspectives*, eds Saiegh-Haddad E. Joshi R. M. (Dordrecht: Springer Netherlands), 31–54. 10.1007/978-94-017-8545-7_2

[B15] Boudelaa S. Perea M. Carreiras M. (2020). Matrices of the frequency and similarity of Arabic letters and allographs. *Behav. Res. Methods* 52 1893–1905. 10.3758/s13428-020-01353-z 32077081

[B16] Carrasco M. (2011). Visual attention: The past 25 years. *Vis. Res.* 51 1484–1525. 10.1016/j.visres.2011.04.012 21549742 PMC3390154

[B17] Carreiras M. Perea M. Gil-Lopez C. Abu Mallouh R. Salillas E. (2013). Neural correlates of visual versus abstract letter processing in Roman and Arabic scripts. *J. Cogn. Neurosci.* 25 1975–1985. 10.1162/jocn_a_00438 23806176 PMC3837287

[B18] Coltheart M. Rastle K. Perry C. Langdon R. Ziegler J. C. (2001). DRC: A dual route cascaded model of visual word recognition and reading aloud. *Psychol. Rev.* 108 204–256. 10.1037//0033-295X.108.1.204 11212628

[B19] Dai J. Ibrahim R. Share D. L. (2013). The influence of orthographic structure on printed word learning in Arabic. *Writ. Syst. Res.* 5 189–213. 10.1080/17586801.2013.827563

[B20] Daniels P. T. (2013). “The Arabic writing system,” in *The oxford handbook of arabic linguistics*, ed. Owens J. (Oxford: Oxford University Press), 412–432. 10.1093/oxfordhb/9780199764136.013.0018

[B21] Decker S. L. Roberts A. M. Englund J. A. (2013). Cognitive predictors of rapid picture naming. *Learn. Individ. Dif.* 25 141–149. 10.1016/j.lindif.2013.03.009

[B22] Dehaene S. Cohen L. Sigman M. Vinckier F. (2005). The neural code for written words: A proposal. *Trends Cogn. Sci.* 9 335–341. 10.1016/j.tics.2005.05.004 15951224

[B23] Deutsch A. Velan H. Michaly T. (2018). Decomposition in a non-concatenated morphological structure involves more than just the roots: Evidence from fast priming. *Quart. J. Exp. Psychol.* 71 85–92. 10.1080/17470218.2016.1250788 27759501

[B24] Duncan L. (2018). Language and reading: The role of morpheme and phoneme awareness. *Curr. Dev. Disord. Rep.* 5 226–234. 10.1007/s40474-018-0153-2 30524927 PMC6244603

[B25] Ehri L. C. Nunes S. R. Willows D. M. Schuster B. V. Yaghoub-Zadeh Z. Shanahan T. (2001). Phonemic awareness instruction helps children learn to read: Evidence from the National Reading Panel’s meta-analysis. *Read. Res. Q.* 36, 250–287. 10.1598/RRQ.36.3.2

[B26] Eviatar Z. Ibrahim R. (2004). Morphological and orthographic effects on hemispheric processing in Arabic reading. *Read. Writ.* 17 691–705. 10.1007/s11145-004-2659-8

[B27] Fabiani E. Velay J. L. Younes C. Anton J. L. Nazarian B. Sein J. (2023). Writing letters in two graphic systems: Behavioral and neural correlates in Latin-Arabic biscripters. *Neuropsychologia.* 185:108567. 10.1016/j.neuropsychologia.2023.108567 37084880

[B28] Foulin J. N. (2005). Why is letter-name knowledge such a good predictor of learning to read? *Read. Writ.* 18 129–155. 10.1007/s11145-004-5892-2

[B29] Frey A. Bosse M. L. (2018). Perceptual span, visual span, and visual attention span: Three potential ways to quantify limits on visual processing during reading. *Vis. Cogn.* 26 412–429. 10.1080/13506285.2018.1472163

[B30] Georgiou G. K. Parrila R. (2020). What mechanism underlies the rapid automatized naming-reading relation? *J. Exp. Child Psychol.* 194:104840. 10.1016/j.jecp.2020.104840 32172942

[B31] Georgiou G. K. Aro M. Liao C. H. Parrila R. (2016). Modeling the relationship between rapid automatized naming and literacy skills across languages varying in orthographic consistency. *J. Exp. Child Psychol.* 143 48–64. 10.1016/j.jecp.2015.10.017 26615467

[B32] Georgiou G. K. Parrila R. Papadopoulos T. C. (2008). Predictors of word decoding and reading fluency across languages varying in orthographic consistency. *J. Educ. Psychol.* 100 566–580. 10.1037/0022-0663.100.3.566

[B33] Ginestet E. Phénix T. Diard J. Valdois S. (2019). Modeling the length effect for words in lexical decision: The role of visual attention. *Vis. Res.* 159 10–20. 10.1016/j.visres.2019.03.003 30904615

[B34] Ginestet E. Valdois S. Diard J. (2022). Probabilistic modeling of orthographic learning based on visual attention dynamics. *Psychon. Bull. Rev.* 29 1649–1672. 10.3758/s13423-021-02042-4 35318586

[B35] Grainger J. Rey A. Dufau S. (2008). Letter perception: From pixels to pandemonium. *Trends Cogn. Sci.* 12 381–387. 10.1016/j.tics.2008.06.006 18760658

[B36] Hansen G. F. (2014). “Word recognition in Arabic: Approaching a language-specific reading model,” in *Handbook of Arabic literacy: Insights and perspectives*, eds Saiegh-Haddad E. Joshi R. M. (Dordrecht: Springer Netherlands), 55–76. 10.1007/978-94-017-8545-7_3

[B37] Hassanein E. E. Johnson E. S. Ibrahim S. Alshaboul Y. (2023). What predicts word reading in Arabic? *Front. Psychol.* 14:1077643. 10.3389/fpsyg.2023.1077643 37187567 PMC10176086

[B38] Hayes A. F. Coutts J. J. (2020). Use omega rather than Cronbach’s alpha for estimating reliability. *Common Methods Meas.* 14 1–24. 10.1080/19312458.2020.1718629

[B39] Henry G. Abou Melhem N. Fiani R. (in press). *BELEA batterie d’evaluation du langage ecrit en arabe [BELEA Arabic written language assessment battery].* Liban: Université Saint-Joseph de Beyrouth, Institut Supérieur d’orthophonie. French.

[B40] Ibrahim R. (2015). How does rapid automatized naming (RAN) correlate with measures of reading fluency in Arabic. *Psychology* 6 269–276. 10.4236/psych.2015.63027

[B41] Ibrahim R. Eviatar Z. Aharon-Peretz J. (2002). The characteristics of Arabic orthography slow its processing. *Neuropsychology* 16 322–326. 10.1037/0894-4105.16.3.322 12146679

[B42] Ibrahim R. Eviatar Z. Aharon-Peretz J. (2007). Metalinguistic awareness and reading performance: A cross language comparison. *J. Psycholinguistic Res.* 36 297–317. 10.1007/s10936-006-9046-3 17318435

[B43] Khan A. Z. Prost-Lefebvre M. Salemme R. Blohm G. Rossetti Y. Tilikete C. (2016). The attentional fields of visual search in simultagnosia and healthy individuals: How objet and space attention interact. *Cereb. Cortex* 26 1242–1254. 10.1093/cercor/bhv059 25840422

[B44] Kim Y. S. Petscher Y. Foorman B. R. Zhou C. (2010). The contributions of phonological awareness and letter-name knowledge to letter-sound acquisition—a cross-classified multilevel model approach. *J. Educ. Psychol.* 102 313–326. 10.1037/a0018449

[B45] Laberge D. Samuels S. J. (1974). Toward a theory of automatic information processing in reading. *Cogn. Psychol.* 6 293–323. 10.1016/0010-0285(74)90015-2

[B46] Lallier M. Abu Mallouh R. Mohammed A. M. Khalifa B. Perea M. Carreiras M. (2018). Does the visual attention span play a role in reading in Arabic? *Sci. Stud. Read.* 22 181–190. 10.1080/10888438.2017.1421958

[B47] Landerl K. Freudenthaler H. H. Heene M. De Jong P. F. Desrochers A. Manolitsis G. (2019). Phonological awareness and rapid automatized naming as longitudinal predictors of reading in five alphabetic orthographies with varying degrees of consistency. *Sci. Stud. Read.* 23 220–234. 10.1080/10888438.2018.1510936

[B48] Layes S. Bouakkaz T. (2022). Predicting word and pseudoword reading in Arabic-speaking children: The independent contributions of phonological and morphological awareness and visual attention. *Br. J. Special Educ.* 49 230–260. 10.1111/1467-8578.12403

[B49] Layes S. Lalonde R. Rebaï M. (2017). Study on morphological awareness and rapid automatized naming through word reading and comprehension in normal and disabled reading Arabic-speaking children. *Read. Writ. Quart.* 33 123–140. 10.1080/10573569.2015.1105763

[B50] Layes S. Lalonde R. Rebai M. (2023). Visuo-spatial abilities and phonological awareness as predictors of reading accuracy in Arabic children with and without dyslexia. *Int. J. Disability Dev. Educ.* 70 1024–1040. 10.1080/1034912X.2021.1952936

[B51] Lervåg A. Hulme C. (2009). Rapid automatized naming (RAN) taps a mechanism that places constraints on the development of early reading fluency. *Psychol. Sci.* 20 1040–1048. 10.1111/j.1467-9280.2009.024 19619178

[B52] Liu J. Ren X. Wang Y. Zhao J. (2023). Visual attention span capacity in developmental dyslexia: A meta-analysis. *Res. Dev. Disabil.* 135:104465. 10.1016/j.ridd.2023.104465 36867955

[B53] Lobier M. Peyrin C. Le Bas J. F. Valdois S. (2012). Pre-orthographic character string processing and parietal cortex: A role for visual attention in reading? *Neuropsychologia* 50 195–204. 10.1016/j.neuropsychologia.2012 22659111

[B54] Lobier M. Peyrin C. Pichat C. Le Bas J. F. Valdois S. (2014). Visual processing of multiple elements in the dyslexic brain: Evidence for a superior parietal dysfunction. *Front. Hum. Neurosci.* 8:479. 10.3389/fnhum.2014.00479 25071509 PMC4083222

[B55] Makhoul B. (2017). Moving beyond phonological awareness: The role of phonological awareness skills in Arabic reading development. *J. Psycholinguist Res.* 46 469–480. 10.1007/s10936-016-9447-x 27535034

[B56] Mansour-Adwan J. Khateb A. Shalhoub-Awwad Y. Cohen-Mimran R. (2023). The different linguistic profiles in Arabic speaking kindergarteners and relation to emergent literacy. *Read. Writ.* 36 2577–2603. 10.1007/s11145-022-10400-4

[B57] McClelland J. L. Rumelhart D. E. (1981). An interactive activation model of context effects in letter perception: Part 1. an account of basic findings. *Psychol. Rev.* 88 375–407. 10.1037/0033-295X.88.5.375 7058229

[B58] Melby-Lervåg M. Lyster S. A. H. Hulme C. (2012). Phonological skills and their role in learning to read: A meta-analytic review. *Psychol. Bull.* 138 322–352. 10.1037/a0026744 22250824

[B59] Míguez-Álvarez C. Cuevas-Alonso M. Saavedra Á (2022). Relationships between phonological awareness and reading in Spanish: A meta-analysis. *Lang Learn.* 72 113–157. 10.1111/lang.12471

[B60] Pelli D. G. Burns C. W. Farell B. Moore-Page D. C. (2006). Feature detection and letter identification. *Vis. Res.* 46 4646–4674. 10.1016/j.visres.2006.04.023 16808957

[B61] Perry C. Long H. (2022). What is going on with visual attention in reading and dyslexia? A critical review of recent studies. *Brain Sci.* 12:87. 10.3390/brainsci12010087 35053830 PMC8773944

[B62] Perry C. Ziegler J. C. Zorzi M. (2007). Nested incremental modeling in the development of computational theories: The CDP+ model of reading aloud. *Psychol. Rev.* 114 273–315. 10.1037/0033-295X.114.2.273 17500628

[B63] Peyrin C. Démonet J. F. Baciu M. LeBas J. F. Valdois S. (2011). Superior parietal lobe dysfunction in a homogeneous group of dyslexic children with a single visual attention span disorder. *Brain Lang.* 118 128–138. 10.1016/j.bandl.2010.06.005 20739053

[B64] Phénix T. Ginestet E. Valdois S. Diard J. (2025). Visual attention matters during word recognition: A Bayesian modeling approach. *Psychon. Bull. Rev.* 32 1165–1203. 10.3758/s13423-024-02591-4 39777606

[B65] Pritchard S. C. Coltheart M. Marinus E. Castles A. (2018). A computational model of the Self-Teaching Hypothesis based on the dual-route cascaded model of reading. *Cogn. Sci.* 42 722–770. 10.1111/cogs.12571 29566266

[B66] Raven J. (2000). The Raven’s progressive matrices: Change and stability over culture and Time. *Cogn. Psychol.* 41, 1–48. 10.1006/cogp.1999.0735 10945921

[B67] Saiegh-Haddad E. Henkin-Roitfarb R. (2014). “The structure of Arabic language and orthography,” in *Handbook of Arabic literacy: Insights and perspectives*, eds Saiegh-Haddad E. Joshi R. M. 3–28. 10.1007/978-94-017-8545-7_1

[B68] Saiegh-Haddad E. Schiff R. (2016). The impact of diglossia on voweled and unvoweled word reading in Arabic: A developmental study from childhood to adolescence. *Sci. Stud. Read.* 20 311–324. 10.1080/10888438.2016.1180526

[B69] Saiegh-Haddad E. Taha H. (2017). The role of morphological and phonological awareness in the early development of word spelling and reading in typically developing and disabled Arabic readers. *Dyslexia* 23 345–371. 10.1002/dys.1572 29154451

[B70] Saiegh-Haddad E. Shahbari-Kassem A. Schiff R. (2020). Phonological awareness in Arabic: The role of phonological distance, phonological-unit size, and SES. *Read. Writ.* 33 1649–1674. 10.1007/s11145-020-10019-3

[B71] Seyll L. Wyckmans F. Content A. (2020). The impact of graphic motor programs and detailed visual analysis on letter-like shape recognition. *Cognition* 205:104443. 10.1016/j.cognition.2020.104443 32882469

[B72] Shapiro L. R. Carroll J. M. Solity J. E. (2013). Separating the influences of prereading skills on early word and nonword reading. *J. Exp. Child Psychol.* 116 278–295. 10.1016/j.jecp.2013.05.011 23892335

[B73] Share D. L. (1999). Phonological recoding and orthographic learning: A direct test of the self-teaching hypothesis. *J. Exp. Child Psychol.* 72 95–129. 10.1006/jecp.1998.2481 9927525

[B74] Share D. L. (2025). Blueprint for a universal theory of learning to read: The combinatorial model. *Read. Res. Quart.* 60:e603. 10.1002/rrq.603

[B75] Steinhilber A. Diard J. Ginestet E. Valdois S. (2023). Visual attention modulates the transition from fine-grained, serial processing to coarser-grained, more parallel processing: A computational modeling study. *Vis. Res.* 207:108211. 10.1016/j.visres.2023.108211 36990012

[B76] Taibah N. J. Haynes C. W. (2011). Contributions of phonological processing skills to reading skills in Arabic-speaking children. *Read. Writ.* 24 1019–1042. 10.1007/s11145-010-9273-8

[B77] Taouk M. Coltheart M. (2004). The cognitive processes involved in learning to read in Arabic. *Read. Writ.* 17 27–57. 10.1023/B:READ.0000013831.91795.ec

[B78] Thompson P. A. Hulme C. Nash H. M. Gooch D. Hayiou-Thomas E. Snowling M. J. (2015). Developmental dyslexia: Predicting individual risk. *J. Child Psychol. Psychiatry* 56 976–987. 10.1111/jcpp.12412 25832320 PMC4672694

[B79] Tibi S. Kirby J. R. (2018). Investigating phonological awareness and naming speed as predictors of reading in Arabic. *Sci. Stud. Read.* 22 70–84. 10.1080/10888438.2017.1340948

[B80] Tibi S. Edwards A. A. Schatschneider C. Lombardino L. J. Kirby J. R. Salha S. H. (2021). IRT analyses of Arabic letter knowledge in kindergarten. *Read. Writ.* 34 791–816. 10.1007/s11145-020-10086-6

[B81] Treisman A. Gelade G. (1980). A feature-integration theory of attention. *Cogn. Psychol.* 12 97–136. 10.1016/0010-0285(80)90005-5 7351125

[B82] Valdois S. (2022). The visual-attention span deficit in developmental dyslexia: Review of evidence for a visual-attention-based deficit. *Dyslexia* 28 397–415. 10.1002/dys.1724 35903834

[B83] Valdois S. Lassus-Sangosse D. Lallier M. Moreaud O. Pisella L. (2019b). What bilateral damage of the superior parietal lobes tells us about visual attention disorders in developmental dyslexia. *Neuropsychologia* 130 78–91. 10.1016/j.neuropsychologia.2018.08.001 30098328

[B84] Valdois S. Reilhac C. Ginestet E. Bosse M. L. (2021). Varieties of cognitive profiles in poor readers: Evidence for a VAS-impaired subtype. *J. Learn. Disabil.* 54 221–233. 10.1177/0022219420961332 32985335

[B85] Valdois S. Roulin J. L. Bosse M. L. (2019a). Visual attention modulates reading acquisition. *Vis. Res.* 165 152–161. 10.1016/j.visres.2019.10.011 31751900

[B86] Verhoeven L. Perfetti C. (2022). Universals in learning to read across languages and writing systems. *Sci. Stud. Read.* 26 150–164. 10.1080/10888438.2021.1938575

[B87] Versteegh K. (2014). *The Arabic language.* Edinburgh: Edinburgh University Press.

[B88] Wagner D. A. Spratt J. E. (1993). Arabic orthography and reading acquisition. *Adv. Psychol.* 103 229–244. 10.1016/S0166-4115(08)61665-9

[B89] Wolfe J. M. (2003). Moving towards solutions to some enduring controversies in visual search. *Trends Cogn. Sci.* 7 70–76. 10.1016/S1364-6613(02)00024-4 12584025

[B90] Zebib R. Henry G. Messarra C. Hreich E. K. Khomsi A. (2019). ELO-L: A norm-referenced language screening test for 3 to 8-year-old Lebanese children. *Arabic J. Appl. Linguistics* 4 24–53.

[B91] Ziegler J. C. Goswami U. (2005). Reading acquisition, developmental dyslexia, and skilled reading across languages: A psycholinguistic grain size theory. *Psychol. Bull.* 131 3–29. 10.1037/0033-2909.131.1.3 15631549

[B92] Ziegler J. C. Perry C. Zorzi M. (2014). Modelling reading development through phonological decoding and self-teaching: Implications for dyslexia. *Philos. Trans. R. Soc. B Biol. Sci.* 369:20120397. 10.1098/rstb.2012.0397 24324240 PMC3866426

