# Supplementary material for: The influence of visual attention on letter recognition and reading acquisition in Arabic
Source: Front Psychol. 2025 Oct 17;16:1628051. doi: 10.3389/fpsyg.2025.1628051 (PMC12576706; doi:10.3389/fpsyg.2025.1628051)
Supplement: Supplementary file 1 [file Table_1.docx]

**Supplementary Material**

**Table S1.**

*Median (Mdn), Minimum (Min.), Maximum (Max.), Skewness (Skew.) and Kurtosis (Kurt.) after data transformation and normalization for each the variables used in the analysis.*

| Variable | Mdn | Min. | Max. | Skew. | Kurt. |
| --- | --- | --- | --- | --- | --- |
| Raven | -0.04 | -2.64 | 3.26 | 0.48 | 1.18 |
| Vocab | 0.12 | -2.11 | 2.35 | 0.07 | -0.66 |
| RAN | 0.04 | -2.22 | 2.55 | -0.00 | -0.39 |
| LK | 0.23 | -2.46 | 1.47 | -0.56 | -0.67 |
| PA | -0.02 | -2.35 | 2.78 | 0.36 | -0.03 |
| LLIT | 0.17 | -2.43 | 1.73 | -0.22 | -1.01 |
| VAS | -0.06 | -2.43 | 1.96 | -0.09 | -0.50 |
| ReadSyll | -0.24 | -1.62 | 3.43 | 0.87 | 0.62 |
| ReadWords | -0.09 | -1.52 | 3.10 | 1.01 | 0.79 |

**Table S2.**

*Pearson Correlations (above the diagonal) and partial correlations (below the diagonal) after control of Raven.*

|  | 2 | 3 | 4 | 4a | 4b | 4c | 5 | 5a | 5b | 6 | 7 | 7a | 7b | 8 | 9 | 9a | 9b |
| --- | --- | --- | --- | --- | --- | --- | --- | --- | --- | --- | --- | --- | --- | --- | --- | --- | --- |
| 1 - Raven | .14 | .25 | .21 | .14 | .22 | .23 | .27 | .13 | .33 | .15 | .38^*^ | .41^**^ | .25 | .33 | .31 | .28 | .31 |
| 2 - Vocab | - | .28 | .22 | .19 | .25 | .16 | .01 | .06 | .02 | .12 | .09 | .12 | .03 | .19 | .19 | .12 | .23 |
| 3 - RAN | .25 | - | .19 | .17 | .24 | .12 | .32 | .12 | .35 | .06 | .26 | .21 | .26 | .35 | .35 | .33 | .35 |
| 4 - LK | .19 | .15 | - | .95^***^ | .96^***^ | .92^***^ | .33 | .18 | .39* | .28 | .59^***^ | .44^**^ | .66^***^ | .79^***^ | .74^***^ | .68^***^ | .76^***^ |
| 4a - LetName | .18 | .14 | .95^***^ | - | .87^***^ | .82^***^ | .26 | .08 | .33 | .27 | .58^***^ | .42^**^ | .66^***^ | .72^***^ | .66^***^ | .60^***^ | .68^***^ |
| 4b - AllogName | .23 | .20 | .95^***^ | .87^***^ | - | .82^***^ | .34 | .22 | .39* | .25 | .56^***^ | .42^**^ | .62^***^ | .81^***^ | .77^***^ | .72^***^ | .79^***^ |
| 4c - AllogDes | .14 | .06 | .92^***^ | .82^***^ | .81^***^ | - | .34 | .20 | .40^*^ | .27 | .54^***^ | .40^**^ | .60^***^ | .71^***^ | .65^***^ | .61^***^ | .66^***^ |
| 5 - PA | -.03 | .27 | .29 | .24 | .30 | .30 | - | .67^***^ | .89^***^ | .31 | .43^**^ | .36 | .42^**^ | .50^***^ | .58^***^ | .59^***^ | .53^***^ |
| 5a - SylSeg | .05 | .09 | .15 | .06 | .20 | .18 | .66^***^ | - | .36 | .20 | .24 | .22 | .21 | .25 | .37* | .39^*^ | .31 |
| 5b - SylDel | -.03 | .29 | .35 | .30 | .34 | .35 | .88^***^ | .34 | - | .22 | .46^***^ | .38^*^ | .45^***^ | .53^***^ | .58^***^ | .57^***^ | .56^***^ |
| 6 - LLIT | .11 | .03 | .26 | .25 | .23 | .24 | .28 | .19 | .19 | - | .40^*^ | .35 | .36 | .29 | .25 | .20 | .27 |
| 7 - VAS | .04 | .18 | .57^***^ | .57^***^ | .53^***^ | .50^***^ | .37^*^ | .21 | .38^*^ | .37* | - | .93^***^ | .87^***^ | .53^***^ | .58^***^ | .51^***^ | .59^***^ |
| 7a - PartielRep | .07 | .12 | .39* | .40* | .37 | .34 | .28 | .18 | .29 | .33 | .91^***^ | - | .62^***^ | .37* | .43^**^ | .38^*^ | .43^**^ |
| 7b - GlobalRep | 0 | .21 | .64^***^ | .65^***^ | .59^***^ | .58^***^ | .38^*^ | .19 | .40^*^ | .34 | .87^***^ | .59^***^ | - | .61^***^ | .64^***^ | .57^***^ | .66^***^ |
| 8 - ReadSyll | .16 | .29 | .78^***^ | .72^***^ | .80^***^ | .69^***^ | .45^***^ | .22 | .48^***^ | .26 | .46^***^ | .27 | .58^***^ | - | .84^***^ | .78^***^ | .85^***^ |
| 9 - ReadWords | .15 | .30 | .72^***^ | .65^***^ | .76^***^ | .62^***^ | .54^***^ | .35 | .54^***^ | .22 | .52^***^ | .34 | .61^***^ | .82^***^ | - | .96^***^ | .96^***^ |
| 9a - Mono | .08 | .28 | .66^***^ | .59^***^ | .70^***^ | .58^***^ | .56^***^ | .38* | .53^***^ | .17 | .45^***^ | .30 | .53^***^ | .76^***^ | .96^***^ | - | .87^***^ |
| 9b - Multi | .20 | .30 | .74^***^ | .68^***^ | .78^***^ | .63^***^ | .49^***^ | .29 | .51^***^ | .24 | .53^***^ | .35 | .63^***^ | .83^***^ | .96^***^ | .86^***^ | - |

*Note.* ^*^ *p* < .05 ; ^**^ *p* < .01 ; ^***^ *p* < .001; *p-values are adjusted using Bonferroni correction for 289 tests*

**Table S3a.**

*Results of Linear Regressions with Syllable Reading as the Dependent Variable*

|  | *Regression 1a (without LK)* | | | | | |  | *Regression 2a (with LK)* | | | | | |  |
| --- | --- | --- | --- | --- | --- | --- | --- | --- | --- | --- | --- | --- | --- | --- |
|  | *b* | *SE* | *b^*^* | *t* | *p* | *R_p_^2^* |  | *b* | *SE* | *b^*^* | *t* | *p* | *R_p_^2^* | |
| (Intercept) | -2.39 | 0.74 | -0.00 | -3.22 | .002** | 0.10 |  | -4.14 | 0.54 | -0.00 | -7.71 | < .001^***^ | 0.39 | |
| Raven | 0.04 | 0.04 | 0.09 | 1.00 | .320 | 0.01 |  | 0.05 | 0.03 | 0.11 | 1.85 | .067 | 0.04 | |
| LLIT | 0.00 | 0.00 | 0.06 | 0.66 | .513 | 0.01 |  | 0.00 | 0.00 | 0.03 | 0.50 | .615 | 0.00 | |
| RAN | 30.00 | 16.43 | 0.16 | 1.83 | .071 | 0.03 |  | 25.78 | 11.29 | 0.14 | 2.28 | .025* | 0.05 | |
| PA | 0.71 | 0.24 | 0.28 | 2.99 | .004** | 0.09 |  | 0.55 | 0.16 | 0.21 | 3.37 | .001^**^ | 0.11 | |
| VAS | 0.03 | 0.01 | 0.31 | 3.21 | .002** | 0.10 |  | -0.01 | 0.01 | -0.07 | -0.95 | .343 | 0.01 | |
| LK |  |  |  |  |  |  |  | 0.08 | 0.01 | 0.71 | 10.37 | < .001^***^ | 0.53 | |

***Regression 1a***: *R^2^* = .37, *F*((5, 96) = 12.83, *p* < .001; ***Regression 2a***: *R^2^* = .70, *F*(6, 94) = 40.63, *p* < .001.

**Table S3b.**

*Results of Linear Regressions with Word Reading as the Dependent Variable*

|  | *Regression 1b (without LK)* | | | | | |  | *Regression 2b (with LK)* | | | | | |  |
| --- | --- | --- | --- | --- | --- | --- | --- | --- | --- | --- | --- | --- | --- | --- |
|  | *b* | *SE* | *b^*^* | *t* | *p* | *R_p_^2^* |  | *b* | *SE* | *b^*^* | *t* | *p* | *R_p_^2^* | |
| (Intercept) | -1.74 | 0.45 | -0.00 | -3.84 | < .001^***^ | 0.13 |  | -2.67 | 0.37 | -0.00 | -7.20 | < .001^***^ | 0.36 | |
| Raven | 0.01 | 0.02 | 0.04 | 0.53 | .600 | 0.00 |  | 0.02 | 0.02 | 0.06 | 0.98 | .328 | 0.01 | |
| LLIT | -0.00 | 0.00 | -0.03 | -0.37 | .713 | 0.00 |  | -0.00 | 0.00 | -0.05 | -0.82 | .414 | 0.01 | |
| RAN | 15.57 | 10.00 | 0.12 | 1.56 | .123 | 0.03 |  | 13.34 | 7.76 | 0.11 | 1.72 | .089 | 0.03 | |
| PA | 0.64 | 0.14 | 0.38 | 4.41 | < .001^***^ | 0.17 |  | 0.56 | 0.11 | 0.33 | 4.92 | < .001^***^ | 0.20 | |
| VAS | 0.03 | 0.01 | 0.38 | 4.19 | < .001^***^ | 0.16 |  | 0.01 | 0.01 | 0.07 | 0.83 | .407 | 0.01 | |
| LK |  |  |  |  |  |  |  | 0.04 | 0.01 | 0.57 | 7.98 | < .001^***^ | 0.40 | |

***Regression 1b***: *R^2^* = .46, *F*(5, 95) = 18.01, *p* < .001; ***Regression 2b***: *R^2^* = .67, *F*(6, 94) = 35.51, *p* < .001.

**Table S4a.**

*Parameters of the structural equation model to predict syllable reading fluency*

| Path |  |  | est | se | *z* | *p* | 95% CI |
| --- | --- | --- | --- | --- | --- | --- | --- |
| RAN | → | ReadWords | 0.11 | 0.06 | 1.81 | .071 | [-0.01, 0.22] |
| PA | → | ReadWords | 0.33 | 0.06 | 5.23 | < .001*** | [0.21, 0.45] |
| VAS | → | ReadWords | 0.07 | 0.07 | 0.91 | .363 | [-0.08, 0.21] |
| LLIT | → | ReadWords | -0.05 | 0.06 | -0.93 | .351 | [-0.16, 0.06] |
| Raven | → | ReadWords | 0.06 | 0.06 | 1.11 | .266 | [-0.05, 0.17] |
| VAS | → | LK | 0.58 | 0.08 | 7.23 | < .001*** | [0.42, 0.74] |
| LK | → | ReadWords | 0.57 | 0.07 | 8.32 | < .001*** | [0.44, 0.70] |
| VAS | → LK → | ReadWords | 0.33 | 0.06 | 5.46 | < .001*** | [0.21, 0.45] |

***Model Fit Statistics:*** 𝜒^2^(2) = 1.23, *p* = .54; *CFI* = 1; *TLI* = 1; *RMSEA* = 0, *p* = .62; *SRMR* = .02

**Table S4b.**

*Parameters of the structural equation model to predict word reading fluency*

| Path |  |  | est | se | *z* | *p* | 95% CI |  |
| --- | --- | --- | --- | --- | --- | --- | --- | --- |
| RAN | | → | ReadSyll | 0.13 | 0.06 | 2.37 | .018* | [0.02, 0.25] |
| PA | | → | ReadSyll | 0.21 | 0.06 | 3.51 | < .001*** | [0.09, 0.34] |
| VAS | | → | ReadSyll | -0.07 | 0.07 | -0.98 | .329 | [-0.22, 0.07] |
| LLIT | | → | ReadSyll | 0.03 | 0.06 | 0.52 | .601 | [-0.08, 0.14] |
| Raven | | → | ReadSyll | 0.11 | 0.06 | 1.92 | .055 | [-0.00, 0.22] |
| VAS | | → | LK | 0.58 | 0.09 | 6.22 | < .001*** | [0.40, 0.76] |
| LK | | → | ReadSyll | 0.71 | 0.07 | 10.82 | < .001*** | [0.58, 0.83] |
| VAS | | → LK → | ReadSyll | 0.41 | 0.08 | 5.39 | < .001*** | [0.26, 0.56] |

***Model Fit Statistics:*** 𝜒^2^(2) = 1.23, *p* = .54; *CFI* = 1; *TLI* = 1.03; *RMSEA* = 0, *p* = .62; *SRMR* = .02
